# Enablers and Barriers to Alternative Care: Perspectives of Community‐Dwelling Older Adults and Service Providers in Kerala

**DOI:** 10.1111/hex.70507

**Published:** 2025-11-30

**Authors:** Anu Mohan, Teddy Andrews Jaihind Jothikaran, Lena Ashok

**Affiliations:** ^1^ Department of Social and Health Innovation Prasanna School of Public Health, Manipal Academy of Higher Education Manipal India

**Keywords:** alternative care, barriers to care, care enablers, integrated care, left‐behind older adults

## Abstract

**Background:**

Decline of intergenerational co‐residence, risk of functional and emotional deprivations in old age, and the diminishing capacity of families to care in‐person for their older parents have intensified the demand for formal care provisions among left‐behind older adults in migrant households of Kerala. While several attempts have been made to quantify the accessibility and utilisation of geriatric care, there is a dearth of evidence on the realities of alternative care in Kerala. The present study aims to explore the perception of older adults (65+) and service providers (government and non‐governmental) on the systemic enablers and barriers of alternative care in migrant households of Kerala.

**Materials and Methods:**

A qualitative approach using IDIs were employed among 20 left‐behind older adults (65+) and 8 service providers across three districts of Kerala (Pathanamthitta, Thrissur and Kannur), using purpose sampling, and thematic analysis was followed using a deductive approach.

**Results:**

Analysis using the Rainbow Model of Integrated Care revealed that the high cost of care at a micro level limits access and utilisation, while the inactivity of day care homes and their failure to upgrade as resource centres challenge the alternative care at a meso level of operation. The absence of need assessments, top‐down implementation, inappropriate fund allocation, lack of measures to sustain the programme and rigid eligibility norms for inclusion pose challenges in the care avenues, while pain and palliative care community‐based associations and doorstep delivery of essential services facilitate seamless delivery of alternative care.

**Conclusion:**

Alternative care efforts should integrate regional, state and central levels of planning, concrete implementation guidelines, participation of older adults in planning and implementation, and efforts to upgrade day care homes into regional resource centres, along with systematic monitoring and evaluation and multi‐stakeholder governance to realise alternative care for older adults.

**Patient and/or Public Contribution:**

Informal discussions with community‐dwelling older adults and service providers helped in refining research questions and in‐depth interview guides to explore their lived experiences. Participants shared insights on their care experiences, which informed data collection and interpretation. Research findings were reviewed within a subgroup of older adults and service providers to ensure contextual accuracy in reporting the practical enablers and barriers of alternative care as perceived by the stakeholders.

**Abbreviations**
ASHA: Accredited Social Health Activist
AWC: *Anganwadi Centres*
LMIC: low‐ and middle‐income country
LSG: local self‐government
NGO: non‐governmental organisation
OA: older adults
RMIC: Rainbow Model of Integrated Care
SHG: Self‐Help Group
SP: service provider
SRQR: Standards for Reporting Qualitative Research

## 1 Background

The drastic increase in population ageing, weakening family structures and rising life expectancy has led to an increase in the proportion of older adults ageing alone, without adequate familial or community support [1]. While families were regarded as the conventional source of social support and security in old age, evolving care landscapes such as transnational migration of adult children and nuclearisation of families, coupled with women's workforce participation, have eroded the informal safety net, leading to unmet care needs and vulnerabilities in ageing alone [2, 3, 4, 5, 6]. The risk of functional and emotional deprivations and the diminishing capacity of families to provide comprehensive care for older adults has intensified the demand for formal care services. The shift in intergenerational care paradigm has mandated care practices which is largely mediated through state, market and not‐for‐profit organisations as envisaged in the care diamond framework for low‐ and middle‐income countries (LMICs) [7].

The WHO framework on integrated care is defined from a life‐course perspective that extends over a continuum of health promotion, treatment and rehabilitation, including palliative care services, which is coordinated within and beyond the health sector [8]. Considering the alarming rise in the proportion of older adults and their unmet needs, a multi‐faceted approach involving government, non‐governmental organisations (NGOs), community‐based models and the private sector is formulated to address the complex health, economic, social and emotional needs of older adults across diverse care needs. Various government schemes have been implemented to provide dedicated services in preventive and promotive care, optimal rehabilitation, and the promotion of geriatric services in India [9, 10, 11]. However, despite their vast geographic coverage and support to infrastructure development, many of the existing efforts are criticised for their restrictive eligibility criteria, low and stagnated assistance, inadequate home‐based care provisions and a centralised and supply‐oriented approach [12]. These systemic deficits have demanded local self‐governments (LSGs), community‐based approaches, such as *Vayomithram, pakalveedu* and Self‐Help Groups (SHGs), to render effective support through regulated care models for older adults in Kerala [13].

Although several attempts have been made to establish a care framework for older adults, insufficient funding, a lack of trained professionals, and shortcomings in an integrated, digitally inclusive and culturally sensitive care model continue to challenge the unique needs of older adults who age alone [9, 14]. While long‐term care remained crucial, most of the interventions primarily focused on institutional healthcare, often neglecting a community care approach. Despite the cultural preference to ‘age‐in‐place’ [15], existing care discourses are challenged by a remarkable inconsistency in the demand and supply of care provisions [16], leading to deficits at various levels of care. Furthermore, the complex interplay of infrastructural, systemic, human resource and financial factors often strains leveraging public–private partnerships at an operational level, demanding multilevel governance in alternative care for older adults. The lack of a nationwide policy for long‐term care [15] and inadequate multidimensional evaluations of integrated care, which focus on process, structural and patient‐level outcomes [17], warrant complementary efforts across private and public domains to fulfil the unmet care needs in ageing.

Despite growing demand for collaborative care models, limited studies have explored the perceived barriers to effective implementation of decentralised care from the perspectives of service providers and older adults, particularly those addressing the needs of older adults living alone [1, 18]. While there are numerous efforts with overlapping objectives for addressing their care needs, there is a paucity of research to interrogate how these interventions function and why some of them remain underutilised from a stakeholder perspective [19]. Existing studies often quantify the accessibility and utilisation of various programmes through nationwide sample surveys. However, the perceptions of service providers and receivers on the care barriers and facilitators remain unexplored, which is pivotal in strengthening the monitoring and evaluation framework of developmental programmes. Furthermore, there is a significant dearth of data on efforts for long‐term care among older adults in Kerala [20], warranting further research to integrate multilevel governance to complement the familial care in migrant households. Although several attempts have been made to measure healthcare accessibility and utilisation, social care remains underexplored and warrants the integration of an ecological approach with the Rainbow Model of Integrated Care (RMIC), to elicit perceived enablers and barriers to alternative care across micro, meso and macro levels. Moreover, understanding of care burden from a service provider's perspective remains sparse in LMICs, suggesting the need for a focus shift from patient to provider [21, 22].

Thus, the present study aims to explore the systemic barriers and enablers perceived by service providers and older adults who age alone outside multigenerational co‐residence in migrant households of Kerala.

## 2 Materials and Methods

### 2.1 Study Design

A qualitative narrative approach was employed to explore the experiences of service providers who are involved in providing decentralised care for older adults in Kerala. This paper is part of a larger study on perceptions and experiences of left‐behind older adults, their caregivers, and service providers in long‐distance caring. However, the present study uses a subset of data on experiences and perceptions of service providers and older adults on extending alternative care for older adults who are left behind by the out‐migration of their adult children. A narrative design includes stories shared by participants, aiming to explore how participants construct meaning from their lived experiences [23].

### 2.2 Study Setting

The study was conducted across three districts in Kerala: Kannur, Thrissur and Pathanamthitta to get an ideal representation of the aged population in the northern, middle and southern Kerala [24]. Pathanamthitta is widely known as a remittance hub due to high rates of international migration, while Thrissur is considered to have a balance of internal and international migration with the highest number of student migrants, and Kannur is traditionally an early centre for Gulf migration, however, now witnessing a decline in emigration and yet continues to be the emigration hub in Northern Kerala [24, 25]. The Kumbanad region of Koipuram Grama Panchayath, Arimpur Grama Panchayath and Koothuparambu municipality was selected from each district purposively based on geographical locations and demographic features.

### 2.3 Participants and Sampling

The study consisted of 20 older adults (65+) who are living alone or with a spouse due to the outmigration of their adult children and 8 service providers, including LSG representatives, officials of NGOs, autonomous institutions related to local self‐administration and representatives of associations for senior citizens. Service providers are directly or indirectly related to designing or implementing alternative care provisions for older adults who age alone. Participants were recruited using purposive sampling. Characteristics table of the participants is summarised in Tables S1 and S2.

### 2.4 Data Collection

A semi‐structured in‐depth interview (IDI) guide was prepared and then translated into Malayalam [vernacular language]. The protocol and tool were approved by the Institutional Ethics Committee (IEC) of Kasturba Medical College (KMC) and Kasturba Hospital (KH), IEC1:248/2022 dated 27 January 2022. Data collection for phase 1 was performed from April 2023 to August 2023, using a semi‐structured IDI guide. Participants were interviewed after scheduling a time convenient for them in advance. The interviews were recorded after obtaining written informed consent from the participants in accordance with the Declaration of Helsinki [26]. Each interview took around 45–50 min and continued until data saturation, the point at which no new information or insights emerged, indicating further interviews would yield redundant data. After completing every third interview, the research team analysed the codes and categories to identify the new and relevant themes which is emerging from the raw data. A minimum of six older adults were interviewed from each of the three districts, and recruitment was stopped when no new codes emerged from the last three interviews, indicating information redundancy across all three districts and the two participant groups, which included older adults and service providers. Data saturation was decided by monitoring recurring themes, verifying data redundancy and confirming data richness among participants. Participants were asked to share their socio‐demographic details (age, annual income, living arrangement, occupation, health status and frequency of visits by children), perceptions and experiences of receiving formal care while ageing apart from families. The interview guide developed for service providers and older adults includes questions such as ‘*Can you describe the experiences of formal care from a non‐governmental or governmental organisation?’, ‘How do they deliver services?’, ‘Is there any program designed for older adults who age alone?*’ and ‘*What are the challenges you experience in terms of formal services?*’ to gain insights into the perceived systemic barriers in providing and receiving alternative care. Field notes were prepared to capture additional information along with the interview recordings. As outlined by Lincoln and Guba (1985) [27], the rigour and quality of this study were ensured by adhering to the four criteria for trustworthiness: Credibility, transferability, confirmability and dependability. The researcher ensured credibility through triangulation of interviews and field notes gathered during data collection and member checking with eight older adults and four service providers to verify that the researcher had accurately reflected on and reported their findings. Furthermore, transferability was ensured by providing a thick description, which contextualised the study findings. Dependability was ensured through an audit trail where the research process is systematically documented. Additionally, a reflective journal was maintained by the researcher to understand biases and ensure that interpretations remained grounded in participants' narratives, thereby ensuring confirmability throughout the data collection and analysis. Research was conducted in a rigorous setting by member checking, where participants reviewed and validated the findings. The researcher maintained a reflective journal throughout the study to understand biases and ensure that interpretations remained grounded in participants' narratives. The SRQR (Standards for Reporting Qualitative Research) was adopted to enhance consistency, transparency and rigour in presenting the findings [28].

### 2.5 Data Analysis

The interviews were conducted in Malayalam (regional language), and the verbatim transcripts were subsequently translated into English by the lead researcher. Another independent bilingual reviewer cross‐checked the English transcripts against the original Malayalam audio recordings, and discrepancies were addressed through consensus discussion. To facilitate the systematic management and analysis of the interview transcripts and field notes, we utilised Computer Assisted Qualitative Data Analysis Software (CAQDAS), specifically NVivo version 13. A thematic analysis, using both deductive and inductive approaches, was performed to derive deeper insights into the systemic barriers in providing and receiving alternative care in later life among left‐behind older adults in Kerala [29, 30, 31]. A subset of transcripts was independently reviewed by the two academic supervisors, and differences in coding were addressed by revisiting the original transcripts, followed by discussions and consensus decision‐making between the lead researcher and supervisors. Broader themes were generated from sub‐themes and codes, followed by a collective review of the themes to enhance.

## 3 Results

**Figure 1:**
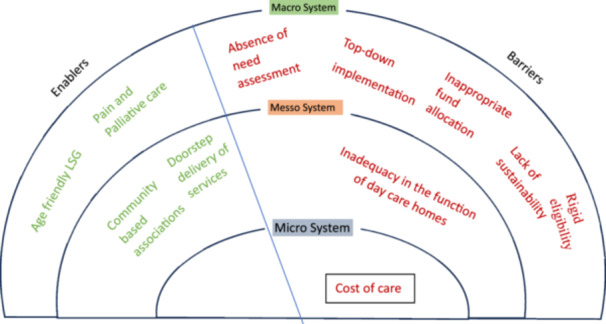
Rainbow of Model of Integrated Care (Adapted).

The inductive and deductive analysis, using the ecological approach and RMIC [32], underscored the multilevel enablers and barriers perceived by service providers and older adults in receiving alternative care provision. RMIC explicitly addresses enablers and barriers across levels (from micro care delivery to macro policy) by depicting the scenarios of fragmentation between health, social and community services among older adults who age alone. The RMIC, developed by Valentijn et al. [33], is a conceptual framework that bridges the scope of care to the level of care integration at the micro, meso and macro levels, which is mediated by enablers and barriers. RMIC integrates person‐centred care at a micro level and population‐based care across meso and macro levels of operation, ensuring a holistic approach in alternative care for older adults. At a micro level, the high cost of care limits access and utilisation, while at the meso level, inadequacy in the functioning of day care homes challenges the quality of alternative care. The absence of need assessments, top‐down implementation, inappropriate fund allocation, lack of measures to sustain the programme and rigid eligibility norms for inclusion pose challenges in the care avenues, while pain and palliative care community‐based associations and doorstep delivery of essential services facilitate seamless delivery of alternative care. Thus, the systemic gaps within the micro, meso and macro levels of interaction underscore the barriers perceived by service providers and recipients in realising alternative care for left‐behind older adults in Kerala. The Adapted RMIC, with major themes unearthed from the analysis, is depicted in Figure 1.

## 4 Conceptual Framework Based on the Adopted RMIC

The RMIC uses the micro, meso and macro levels of integrated care by categorising its different dimensions according to their scale of operation within the larger care system.

### 4.1 Enablers of Alternative Care

### 4.2 Macro‐Level Enablers

RMIC views the macro‐level enablers as the larger structure, comprising funding and regulation of the entire health and social care landscape. Our analysis unearthed age‐friendly LSG and the interdisciplinary care through the wide pain and palliative services as the specific macro‐level enablers of alternative care in migrant households.

#### 4.2.1 Age‐Friendly Local Self‐Governance

Several service providers acknowledge the wide systemic acceptance for elder care, through various organised efforts including dedicated budget, pain and palliative care, day care homes (*pakalveedu*), Special Village Council *(Gramasabha)* and geriatric centres as envisaged in national policies for older adults and an age‐friendly governance framework. One of the service providers stated his perception of special budget allocation and working with older adults in facilitating care for them as follows:

Panchayaths in Kerala allocate 5% of their annual fund for vulnerable categories. Recently, a good amount of the funds has been allocated for the welfare of older adults. There is a gradual shift from ‘work for elders’ to ‘work with elders’ to realise the real needs of the ageing population.

SP1

Similarly, one of the older adults stated his perception on the importance of vayo clubs, special gram sabhas and grassroots planning in enabling geriatric care as follows.

‘Our panchayat has won an award for the commendable initiatives in geriatric care. We have a rigid supportive system where older adults are considered crucial in planning and implementation of welfare agendas. Our health and social conditions are surveyed by the panchayath, and they often reach out to the most vulnerable within the panchayath. We have Vayo clubs, special Village Council (Gramasabhas) to discuss anything that matters to older adults’.

SP4

Besides funding, planning and multi‐sectoral collaboration between ASHA workers, Kerala police and *Anganwadi* workers are deemed to be pivotal in realising seamless care for older adults across varying functional capacities. A few service providers stated as follows.

‘Asha workers, Anganwadi workers and Kudumbasree members have been working actively to implement programs for older people. With the introduction of Vayo Club, the initiative Vigilance Committee (Jagratha Samithi) has been merged, and it works hand in hand’.

SP4

‘One of the highlights of Kerala's elderly policy is to ensure police protection for the elderly living alone by responding to emergencies. For example, bringing the elderly to hospitals in times of need. If there are any emergencies, responses like running for help are expected from the side of the police force. Some use such services efficiently in Thrissur itself’.

SP7

#### 4.2.2 Pain and Palliative Care Services

Within decentralised care efforts, several left‐behind older adults perceived pain and palliative care as a reliable source of alternative care, especially when their mobility declines beyond a limit, demanding in‐person care with Activities of Daily Living (ADL). Several service providers and older adults stated as follows.

‘Palliative care is the prominent service for bedridden older adults. Many have 10 to 15 teams. A team of palliative care workers and palliative nurses visit the elderly at home to ensure necessary medications, tests, and care. Our team is ready to perform tube changes and dressings for inpatients. Similarly, the panchayat is working tirelessly to provide the necessary equipment, including an airbed, coat, walking stick, and diaper’.

SP2

‘We have an active pain and palliative care in our panchayath. If we fall bedridden, their team will come home, do the required routine check‐up up and assist us with devices or services if needed. It's indeed a good initiative, especially for older adults who have mobility issues’.

OA‐13

In addition to the pain and palliative care services operated by LSG institutions, several NGOs are actively engaged in complementing these efforts by providing home‐based care. As noted by an NGO representative:

‘We do have mobile medical units. We have a traveller and a core team comprising a doctor, a social worker, nurses, and a driver cum community mobilizer who renders mobile check‐ups and medical consultations. The team covers 2 locations per day and a total of 10 locations in a week’.

SP1

While there is a huge acceptance for pain and palliative care, efforts to train the workforce are perceived as successful in meeting care needs among left‐behind older adults in migrant households. A few service providers started decentralised assistance and training for caregivers as enablers in alternative care. They stated as follows:

‘The palliative care centre close by is providing training to the care team. If there is a caregiver within the household, we provide them with adequate training so that they can complement our efforts. We targeted households with a readily available caregiver. We have clustered and are trying to work on clusters, each with 50 households. Kudumbasree is also given charge for the same. It's progressing well’.

SP4

### 4.3 Meso‐Level Enablers

RMIC views the meso level as the middle scale of operation, which acts as a bridge between individual care and the overall system, focusing on how various structures and relationships enable micro‐level care. Our analysis has revealed that home‐based delivery of essential services and support from community‐based peer lead associations enhances alternative care and support among older adults in migrant households.

#### 4.3.1 Doorstep Delivery of Essential Services

Besides health support through panchayaths, several older adults seek assistance in getting groceries, vegetables and cooked food delivered to their doorstep, especially when they age alone. A substantial number of participants perceived the importance of a wide range of home‐based services offered through private entities, as stated below,

‘We have a private palliative care service, and they have many services, including mobile health checkups, home‐based consultation of doctors and the collection of blood and other samples for testing. In addition to this, home delivery is also under them, where vegetables and other things are processed and delivered to the home…. Membership holders can get many medical tests done at a low cost. Such services are much needed now’.

OA‐13

Another participant stated the importance of food delivery, which is centrally cooked and delivered to households where older people live alone and struggle to cook for themselves. He mentioned as follows.

'Food delivery systems have already become prevalent. Here we have a system where older adults living alone are delivered to homes as per order, which is cooked centrally. It is a need of the hour. It will be a job for those who cook and sell food, and it will also be a help to elderly people who are struggling to cook food while living alone. In this era of an increasing number of elderly people, such initiatives will be very helpful for the elderly in families who do not have people to cook and serve alone’.

OA‐10

#### 4.3.2 Community‐Based Support

Besides decentralised governance, membership in community groups, senior citizens' forums and religious organisations served as a vital enabler of care. Several participants perceived that such facilities promote access to resources, improve social connections and enhance visibility of members' needs in the community, leading to timely support. One of the male service providers stated as follows.

‘Membership in a community‐based group can be especially beneficial for older adults who are isolated at home. The foundation of any resource centre is this link to a community of like‐minded people, and hence this coming together of older adults itself is a solution to the wide range of emotional issues they experience’.

SP1

While community‐based associations were perceived to play a pivotal role in realising alternative care, one of the regionally formed associations was observed to stand out for its unique vision to combine activities of clubs for older adults with *Anganwadi Centres (AWCs)* to ensure multigenerational integration and sustainability through participation. Another service provider stated his perception of community‐based groups as follows:

‘I formed a voluntary organisation **. A group of 125 families is currently working here. Sumitra is not a system for rehabilitating older adults, but rather a platform for planning and organising various activities for the welfare of the older adults. We created friendships through senior clubs and senior gatherings by mobilising them to solve the problems of others. We have conducted music clubs where people sing, dance, and celebrate every moment together with similar problems. We have 34 Anganwadis and a functional old age club, which operates through Anganwadis. Clubbing older people with Anganwadi has increased the inclusion of older people in community activities and allied areas’.

SP5

Similarly, a few older adults shared their experience of participating in such voluntary organisations as follows.

‘There is a program once a month in our group**. Older people get together to talk for a while; those who like to sing can sing, give speeches, and tell stories. We will find time to read poetry and take Bible quizzes. I work as an organisation treasurer’.

OA‐9

Besides support groups that work not for profit, several older adults perceived religious groups, especially church‐based associations, as a major source of alternative care. Several participants view religious associations as a source of care, spiritual support and emotional resonance. Similar experiences were stated by a male participant and a female participant as follows.

‘Our church is mostly comprised of senior citizens, and we see it as an opportunity for all of us to come together.… Around Christmas, we visited bedridden senior citizens. One or two times a year, we get a chance for such visits. It was a good experience, and they were happy too. Church unites us every Sunday and thereafter for the associational activities. While we live away from our children, having peers to support each other is the biggest joy that cannot be compromised’.

OA‐5

‘One day a month, we gather in church for singing, playing minor games, and group prayer. At least once a year, we go on small picnics with healthy people who can travel. We also take places at least. Spiritual talks and expert‐led classes on healthy ageing are organised by the church itself’.

OA‐4

## 5 Perceived Barriers in Alternative Care

### 5.1 Macro‐Level Barriers

Macro‐level barriers are viewed as the deficits in the overarching political, financial and legal environment that hinder integrated care of a region in a broader socio‐legal context. Our analysis of large‐scale barriers in alternative care has revealed that inadequacies in planning, implementation and designing of programmes, rigid eligibility regulations and skewed funding hinder alternative and integrated care at a systemic level.

#### 5.1.1 Absence of Need Assessment Before Service Delivery

While several decentralised care efforts were found pivotal by several left‐behind older adults, the analysis highlighted multiple barriers to realising alternative care in migrant households. Several service providers reported a lack of proper need assessment before the implementation of welfare programmes as a challenge to their efficacy and utilisation. Another service provider stated the lack of communication, feedback and need‐based planning between beneficiaries and benefactors before making care investments as follows.

‘The basic need is often not considered while formulating government programs. When the government orders to buy of beds for inpatients, we are investing a new resource in a person who is waiting for death. The person may die on the second day of admission. And for whom was that bed bought, and why was that an investment? … who told the government that his greatest necessity is in bed? The local self‐governing bodies then must bridge this communication gap. What the local self‐governing bodies should do is find out what their real needs are and pressure them to get financial assistance from the government'.

SP5

Like the perception of the service provider, several older adults shared the absence of specific programmes for left‐behind older adults that target only the unique needs of older adults who age alone. Several older adults perceive the lack of need assessment as a barrier to realising alternative care

‘Unlike the structured pain and palliative care services for bedridden older adults, no dedicated programs are addressing the needs of older adults who age alone. No one has asked me so far if I have any concerns or special needs while I age alone in my 80s. From my experience, the needs of the beneficiaries are not understood by the one who oversees the program implementation’.

OA‐3

‘Government or non‐governmental organisations never plan anything based on our requirements; they do what they feel like doing, and it is beneficial for them. If they want to spend some money, they do what gets more public attention. It need not be a real need for the one whom they target’.

OA‐1

Additionally, the lack of grassroots planning through decentralised means like Village Council (*Gramasabhas)* often limits decision‐making efficiency, as stated by one of the service providers.

‘The concept of people planning was to form “Ayalkootam” (Neighbourhood Group) for 50 houses. How many neighbourhood groups are active today, even though the intention was that all planning programs should be discussed and formulated based on the decision of the neighbourhood group?… But unfortunately, many gram sabhas are met for name's sake while they are not represented in decentralised planning, resulting in improper need assessment’.

SP5

Several older adults and service providers view a periodic need assessment as a means to increase the utilisation and efficiency of programmes for older adults

‘If the real needs of left behind people should be addressed, at a grassroots level, a population survey, participatory discussions through Gramasabha (Village Council) should be held to brainstorm the felt needs of older adults. Programs should be planned after carefully studying those who are targeted to benefit from it. One program for all might not work considering the uniqueness of issues across rural and urban needs in ageing’.

OA‐9

#### 5.1.2 Top‐Down Implementation and Decision Making

Besides planning issues, various programmes in place for the welfare of older adults are reported to have flaws in implementation, often due to a lack of specialised knowledge among officials. There is a gap between theoretical expertise and practical experience in implementing programmes, as reported by the service provider. One of the service providers stated the absence of adequate understanding among the implementers as the key reason for inefficiency of programmes, as follows:

‘Most of the programs are planned and implemented by people who have no knowledge or experience in these areas. But in such government projects, officials think that they are subject experts. But the fact is that they do not have practical insights about the implementation of the program. They are only trying to bring the knowledge they have read and learned from some sources to a practical level. So often, the problems we are trying to solve are not meeting the feasibility of practical execution’.

SP1

Analogously, few service providers stated how programmes are presented as community‐driven even when the decisions and framing are shaped by bureaucrats or higher authorities. He reported his experience where needs are often assumed without asking them.

‘Whether women are children, disabled or elderly, it is often through the media that they know what their needs are. It is a kind of ventriloquism. If I cannot say what my real need is, there may be no solution. They are never once asked or able to tell what their needs are. Someone else is making decisions and creating programs for them’.

SP7

Similarly, another service provider reported improper delegation, weak monitoring and evaluation as the core reasons for the weak implementation of programmes for older adults.

‘Even though we say doorstep delivery of service, who should provide doorstep services? Is it enough only for bedridden patients? What services should arrive at the doorstep? The goal of all projects is better. But its implementation is not perfect. There are, of course, errors of expression in translating the government's objectives into its working words. I would not say that we need to create many new programs…. Proper delegation, monitoring, and evaluation should be strengthened for existing programs to ensure implementation efficiency’.

SP8

Several participants suggested the need for involving the local leaders, subject experts, retired officials and older adults who are the potential beneficiaries in the planning, implementation and evaluation of programmes that target care of older adults.

‘Programs should be the felt need of the beneficiaries, and hence voices representing the target group, community and older adults who are experienced in administration and implementation should be pooled from the community where the program is planned. Ward members, retired government officials, and older adults with diverse needs and problems should take part in different phases of planning and implementation’.

OA‐13

#### 5.1.3 Inappropriateness of the Fund Allocation

While decentralised planning insists on 5% of the annual plan allocation for the welfare of older adults, there are no speculations on spending the funds allotted, leading to inappropriate fund utilisation, which is beyond the perceived need. Another service provider stated that LSG is keen to spend funds on assistive devices, while there are several other needs which require immediate attention and reported as follows:

‘Even when it says that 5% of the budget should be spent on the elderly, it does not specify how much of the 5% should be spent on daycare homes. Therefore, in such situations, the effort of ward members to answer the question of how to utilise this five per cent will end up with ideas like spectacles, cots, water beds, and assistive devices like wheelchairs’.

SP3

A substantial number of participants described the scenario where the entire fund allotted for older adults is being utilised in pain and palliative care, leaving behind the other requirements in ageing. They reported as follows.

‘The major problem is that a significant percentage of existing funding is allocated to palliative care only. Only a residual amount is considered for other needs of senior citizens. Some needs go beyond physical assistance for bedridden patients. Existing fund utilisation overlooks the social and emotional needs of older people who might not require a waterbed and spectacles’.

SP7

‘I know that 5% of the Panchayath's annual plan is expected to be spent on the welfare of older adults. But so far, we have not received any benefits from it. Most of the funds are being directly used for pain and palliative care, to increase the reach and visibility, without even considering if there is a need for other areas of action, too. I feel the fund allocation is improper, and it needs to be addressed at the earliest’.

OA‐1

#### 5.1.4 Absence of Measures to Sustain Programmes

In addition to implementation issues, new programmes with overlapping objectives, abrupt discontinuity of existing programmes, constant changes in administration and political influences seem to reduce the sustainability and efficiency. One service provider stated the need for continuing existing programmes rather than the frequent introduction of new initiatives with changing political leadership as follows:

‘Any new officer taking charge is thinking about “what can be innovated during their tenure.” In every era, programs are formulated without proper study or analysis, so it is abandoned in their tracks. Therefore, rather than inventing new programs, it is necessary to strengthen the existing programs … What the authorities want is to publicise “what they have started in my time. This immature idea causes many programs that were working well until then to stop and start something new just for the sake of starting. This tendency repeats, and this is the major reason why no program ever fully completes its intended purpose. The same happens when the political party changes and the regime changes'.

SP1

Similarly, one of the older adults stated the challenges in sustaining programmes due to political tension from opposing parties, as stated below.

‘None of the current activities has reached a hype. It is noticed that any program is confined to the bottom or one sector only. Whenever any program is formulated, there are opposing political forces in our state who try to taint the purity of its ideology, be it communal or religious…, activities that suppress its intention will continue to occur from the opposing party, leading to a lack of sustainability in continuity, no matter how good its intention is’.

OA‐10

Besides political tensions, the lack of interest from the beneficiaries and limited participation seem to challenge the continuity of programmes. One of the participants reported as follows.

‘Often, more than a lack of programs, a lack of interest among senior citizens prevents the continuation of many programs…. What I have said is that no matter how many programs are devised, unless people's participation is ensured in them, they will not reach their true purpose. Strategies to get people to be a part of the program should be adopted first. Programs should have elements that feel interesting and needed for the beneficiaries to make it sustainable’.

OA‐10

Additionally, several older adults perceived the inability to create required awareness and sensitisation on services as a major barrier in alternative care utilisation. A few participants stated as follows.

‘While there have been many initiatives in government policy focusing on older people living alone, the concerned departments have not paid enough attention to implementing them in a way that the real beneficiaries feel their importance and usefulness. Whether there are helpline numbers, we should check how many older adults are familiar with these services. Various awareness campaigns have been conducted, but many of them have not fully reached their destination. Many older people don't know they really deserve'.

OA‐11

#### 5.1.5 Rigid Eligibility‐Based Grouping of Older Adults

Besides the need assessment, the services often tend to categorise adults based on age, overlooking individual capacities. One of the service providers highlighted improper categorisation based on age and the absence of person‐centred care plans as a key barrier to availing care as follows.

‘We usually categorise seniors as 60‐70, 70‐80, 80‐90. But this type of classification is fundamentally wrong…. Not all 90‐year‐olds are bedridden. But someone in their 60s can become bedridden. What I am saying is that we need to think about how to ensure services based on a person's fitness rather than their age. The concept is to address ability‐based varied needs…. Therefore, an age‐based categorisation is fundamentally difficult…. Thus, the important agenda is to plan the required services for these sections based on their need’.

SP1

Due to strict categorisations, several programmes were reported to primarily benefit older adults below the poverty line; they often overlook non‐financial needs such as psychosocial support, caregiving services and functional needs. Another service provider stated as follows.

‘The beneficiaries are often not the entire elderly population but certain sections of the population. The beneficiaries of most welfare programs are the very poor and those whose incomes fall below a fixed annual income. Mental health, mobility and support in later life extend beyond income’.

SP5

A similar perception was stated by one of the older adults, stating the risk of exclusion of older adults with limited functional capabilities. Thus, a 77‐year‐old male participant stated the absence of equity measures as one major barrier to accessing health. He stated as follows.

'Many of the interventions for older adults will not cater to those across different functional capabilities. For example, when an older adult falls bedridden, he cannot go and access services in person. The services should reach him rather than him going behind the provisions. Such systems often lack in our care planning, leading to exclusion and deprivations in ageing’.

OA‐9

### 5.2 Meso‐Level Barriers

According to RMIC, the meso‐level barrier comprises the implementation gaps within organisations and structures that provide care services and is positioned between the macro level and micro level of the system. Our analysis has unearthed inadequacies in the functioning of day care homes and their failure to upgrade as regional resource centres as a crucial barrier to accessing alternative care.

#### 5.2.1 Inadequacy in the Functioning of Day Care Homes

While several day care homes are constructed, many of them reported to remain functionally inactive, due to a lack of programmes that cater for the needs and interests of older adults, limited accessibility to the centre, absence of dedicated transportation facilities back and forth and limited care facilities in the centre. One of the service providers reported the inability of care homes to render interesting programmes for older adults, as a barrier to realising alternative care, as stated below.

‘Another option to spend the fund is to build daycare homes for older persons. In general, panchayats are interested in making any such conspicuous expenditure. So, they built a building and a toilet…. Then, the inauguration of the day houses with great publicity will be done with a big board titled “Pakalveedu (day care home)”, but at the end of these efforts, the daycare centres will become stagnant. Will anyone come to the Pakalveedu just because of these buildings and titles? Will anyone come because we put out a few newspapers or buy a TV? They have facilities to watch TV and read newspapers in their own homes. Then, how will we make daycare homes (Pakalveedu) useful for the elderly?’.

SP1

Despite the availability of quality programmes, a few service providers noted that challenges such as accessibility issues, lack of transportation and the absence of dedicated caregivers significantly contributed to the underutilisation and inactivity of daycare centres. Few LSG representatives stated as follows.

‘We have pakalveedu (day care home), but it is closed for now. Because it is quite far from here, and people find it difficult to reach there. We got that place through sponsorship, and hence, the distance and accessibility are still a concern. We cannot blame people for not coming here, as it will cost around 70 Rs auto charge per side. So, practically, the distance is a concern, and hence it is not functional now’.

SP4

‘It must be said that there are daycare centres in the panchayat, but they are not efficient. Even if there are day homes, there is no point in building a mere daycare facility without ensuring transportation facilities for the elderly to reach there. Constructing buildings without ensuring means to access them makes no sense for me’.

SP3

While several service providers perceive the barriers with systemic and infrastructural limitations in accessing care, several older adults stated the lack of engaging programmes, distance to the centre and lack of sustainability in providing quality programmes as a major shortfall in care. Another participant stated as follows.

‘I hardly visit Pakaveedu. First, it is a bit interior, and I cannot walk there. Even public transport is too limited, and I cannot afford to take an auto back and forth just to spend half a day there. Even if I manage to reach there, I can read a newspaper or watch TV for some time, which is possible even at my home. The whole point of the day daycare home seems non‐functional, at least in my place’.

As the distance to *pakalveedu* is noted as a reason that limits its accessibility and utilisation, few older adults have suggested converting nearby public places and houses of common acquittance as a place where older people gather occasionally. One of the service providers stated that such informal associations would be more effective than formal initiatives, which are not accessible.

‘Even rural libraries can act as daycare homes (pakalveedu) if they can be directed properly. There is no need to set aside separate funds for building huge infrastructure. In our community, and houses of each member can be converted into venues for setting up meetings and related events. Many associations without many infrastructural setups already align in this line’.

SP1

Several service providers suggested the urgent need for upgrading *pakalveedu* (day care homes) from a mere day care facility to a resource centre which ensures a range of services, including consultation, regular health checkups, recreation and training to remain functional. One of the service providers stated his perception of inability to upgrade day care homes as follows:

‘Daycare centres (pakalveedu) should be started in all panchayats as an initial phase, and in the coming stages, they should be raised as Regional Resource Centres. The regional resource centres recommended, along with this aim, to ensure services such as physiotherapy, Yoga, meditation, mobile care services, teleconsultation, regular health checkups through doctors, recreation and entertainment, counselling desk, community coordination and outreach, geriatric centres, digital literacy heritage visits, distribution of assistive devices, food, and nutrition, to and transportation. Assistive devices can be rented on a monthly or daily basis. So, this service can be extended as per the requirement. The basic idea is not to splurge on resources overnight, but to upgrade existing daycare homes(pakalveedu) into resource centres of this kind’.

SP1

Another major suggestion to improve the functioning of pakalveedu is to enhance the representation of the older adults themselves in the management of these programmes, as stated by one of the service providers.

‘Often, the main reason why many declarations fail to translate into a practical expression is that the daycare homes (Pakalaveedu) do not emerge as resource centres. That is when the management of day care homes is taken over by the older adults themselves. Instead of the panchayath deciding on the activities of day care homes, older adults who participate should be delegated the authority to take it forward under the supervision of LSGs’.

SP5

### 5.3 Micro‐Level Barrier

The micro level of operation in RMIC is the smallest scale, which is focused on the actual care process and the direct relationship between the care providers and the receivers. Micro‐level barriers thus encapsulate what hinders person‐centred care among older adults who age with limited personal care. Our analysis has uniquely indicated that the high cost of person‐centred and private care often reduces the use of alternative care provisions in migrant households.

#### 5.3.1 Cost of Care Outside the Home

While several service providers perceived systemic challenges like top‐down decision‐making, inappropriate fund allocation and strict eligibility in care, older adults perceived a lack of affordable care measures as a major barrier in assessing alternative care while ageing alone. Several older adults expressed the need for government intervention in regulating paid care services by prioritising support for those who are financially disadvantaged. A few participants perceived a lack of adequate government support as a barrier and stated as follows:

‘Every citizen living in Kerala pays a share of his earnings to the government in the form of taxes. Therefore, as many other countries do, the government must provide many services to ageing citizens either free of charge or at a reasonable cost. Of course, special consideration should be given to the elderly who are isolated and who lack financial security’.

OA‐20

On this occasion of getting an old‐age pension of 1000 or 2000 rupees, common senior citizens cannot even think of external care. As far as I understand, care models and social insurance are there for the elderly in foreign countries, and they are under the control of the government. Similar systems need to be put in place in India.

OA‐19

Similarly, another older adult perceived the absence of government care facilities as a barrier to availing alternative care. Another participant stated as follows:

‘Certain other countries with a similar socio‐economic profile have already implemented old‐age care programs and pensions schemes. If they are not willing to provide a pension government should sponsor the care and maintenance through various means. That is what the rest of the countries do. But in India, we don't have any returns in ageing out of the tax they charge for everything we use’.

OA‐16

Similarly, one of the female participants who is not covered under any pension stated the challenges about the cost of care and lack of subsidised care as a barrier to receiving quality care, as stated below.

‘We can't expect the government to afford all expenses related to care. But it is highly appreciable if the government can interfere in formal caregiving by making it affordable. Lack of Subsidies in care and absence of care consideration or allowances for the neediest is indeed a concern for someone like me who doesn't have a pension’.

OA‐17

Another participant stated the perceived need for the government to bear a part of the care expenses when families cannot afford to pay the entire expense of hiring a caregiver. One of the participants stated as follows.

‘Local self‐governing bodies need to intervene in villages like this, where not everyone is economically equipped to appoint a home nurse. In such a situation, the government should train and create a pool of caregivers who can be recruited to households with strong care needs. This care option should be provided at an affordable price; households and government should bear a stake in care if families themselves cannot afford the care’.

OA‐13

While several participants perceived the affordable and decentralised formal care, several others also perceived that not contributing adequately to the government as tax as a hindrance to receiving quality care while ageing alone. Several participants perceived the absence of social assurance as stated below.

‘While we share many development dreams, we are always compared to Western countries. But no one wants to talk about what percentage of income is paid in taxes in Western countries to provide free elderly care schemes. Therefore, when asking for free services, it is also necessary to think about what kind of steps should be taken to ensure it’.

OA‐8

## 6 Discussion

The present study aimed to explore the perceived systemic enablers and barriers in the implementation of alternative care services for left‐behind older adults in Kerala and the recommendations to scale up new and existing measures. Thematic analysis unearthed four major enablers across the macro and meso levels of operation, along with five macro‐level enablers, a meso‐level enabler and another micro‐level enabler, indicating cross‐level integration of alternative care in migrant households.

## 7 Micro‐Level Enablers and Barriers

RMIC, using micro, meso and macro levels of operation, aims to address the enablers and barriers in delivering alternative care for older adults in migrant households. While our analysis focused on broad systemic perspectives, the analysis using RMIC has unearthed the cost of care outside the home as a major micro‐level constraint to accessing alternative care. Although there is a huge demand for ‘*pay and stay’* care provisions [34], the high cost associated with such facilities often hinders several families beyond the threshold income from availing such services, suggesting the need for more affordable alternative care models. While previous research on a micro‐level care enablers concluded individual factors such as fear of institutionalisation, comorbidity, frailty and help‐seeking patterns, as constraints in care [13, 35, 36, 37, 38], our analysis implies that the cost of care often extends beyond individual capability and presents a broader systemic barrier to alternative care for older adults in migrant households. When the high cost of care is perceived as a micro‐level barrier, effective communication between the care provider and receiver, tailored care planning and a trusting relationship are reported to enable care at a micro level [32]. However, our analysis did not yield any themes under micro‐level enablers of integrated care (RMIC), suggesting the shortfalls in delivering person‐centred care among older adults who age alone. Furthermore, the absence of drivers of micro‐level care when compared to multiple barriers implies that older adults in migrant households primarily experience fragmented and top‐down services, suggesting interventions that target need‐based and shared care planning.

## 8 Meso‐Level Enablers and Barriers

RMIC views meso‐level enablers and barriers as organisational and operational structures that bridge the micro and macro levels of care. As a continuum to micro‐level enablers, community‐based associations for older adults and doorstep delivery of essential services contributed to a meso‐level delivery of alternative care provisions. Previous evidence also argues that community inclusion and peer support are pivotal in compensating for the physical ailments and social dysfunction in growing old [39, 40]. Our analysis further revealed that governmental and private efforts to deliver home‐based services, such as doorstep collection of blood samples, delivery of food and groceries and home‐based medical consultation and services, played a pivotal role for several older adults who age in migrant households with minimal support. While our analysis unearthed the meso‐level support in care, Gustafsson et al. [41] argued the need for more interventions to scale up NGO helplines and home‐based aids for the physical and emotional care of older adults who age alone. In addition to home‐based service delivery, HelpAge India's recreation programmes and health camps were reported to be effective in enhancing the quality of ageing at a meso level of operation [42].

While community‐based organisations are reported to be vital in enabling care, our findings underscore the perceived failure of day care homes in extending care, and the reasons for their perceived inefficiency [3]. Although day care homes *(pakalveedu)* in Kerala are well known for their relevance and infrastructure [43], our analysis yielded contradictory findings, concluding lack of accessibility due to its remote location, the absence of coordinated transportation facilities to and from the day care homes, an inadequately designed programme and limited manpower to oversee its implementation were reported to have reduced the effectiveness of several *pakalveedu* (day care homes) in complementing familial care. Besides reduced accessibility and compromised quality, several day care homes were criticised for their perceived limitations in evolving into regional resource centres where older adults are empowered holistically. While there are several functional day care homes across Kerala, the non‐uniformity in their effective operations could be due to the political and regional variations in decentralised implementation, suggesting a statewide implementation framework and robust monitoring and evaluation mechanisms. While existing evidence highlights a dual stance on the effectiveness of state‐owned day‐care homes, our analysis uncovered a unique success model in Kerala, which is a locally emerged SHG for older adults. This group is empowered through *AWCs* by bringing them together for leisure and support. While such efforts were found crucial in mobilising older adults through grassroots approaches, our findings across three geographical regions of Kerala concluded that such systems are limited to places where local leadership and community support are strong enough to sustain programmes. Similar findings on the differences in community engagement and regional variations in the care demands and satisfaction were reported by Makwana and Elizabeth [36], implying non‐uniformity in community support across rural and urban regions. This rural–urban divide recommends the integration of local stakeholders and programme monitors to improve targeted care efforts to reduce the barriers to inclusion [44].

## 9 Macro‐Level Care Enablers and Barriers

Using RMIC, our analysis explored how the larger system drives and hinders alternative care for older adults in migrant households of Kerala. Macro‐level regulations, policies and systemic factors, such as age‐friendly local self‐governance, dedicated budget for older adults in LSG annual plans, and pain and palliative care programmes for acute pain management, were revealed to be crucial for older adults, who age alone with limited familial support. While age‐friendly LSG is perceived as a macro‐level enabler, as viewed by RMIC, its operation and decentralised implementation spread across meso and macro systems, suggesting the need for an integrated care approach as envisaged in the RMIC. However, Bolt et al. [45] reported the influence of education, autonomy and medication literacy as major care enablers from a health system perspective. While previous evidence highlights the commendable efforts in social security and health care, our analysis exposed the divergence and loopholes in utilising 5% of the annual budget exclusively on geriatric care. Furthermore, the present study revealed the predisposition to allocate resources primarily to pain and palliative care, despite several pressing care needs beyond acute and chronic pain management in ageing. Ineffective financial monitoring and evaluation frameworks [14], which assess the long‐term impact of fiscal responses in social care and political biases to engender visibility of outreach activities, could be the major reasons for the skewed fiscal responses in integrated care, suggesting robust need‐based fund planning in long‐term care for older adults. In line with the findings reported by Indirabhai et al. [46], our analysis integrating RMIC has highlighted that the Kerala model of home‐based palliative care is crucial in delivering quality care and establishing intimate relationships with patients, especially in migrant households. However, Carey et al. [47] reported contradictory findings, where inadequate training awareness, communication issues between healthcare professionals and the absence of a national palliative care policy at a macro level imply dual narrations on home‐based care services in India. However, despite the shortfalls and implementation gaps, Kerala's palliative care policy remains the first policy in Asia and a significant milestone in the palliative care movement, which mandated government interventions in strengthening community‐based palliative care programmes [48]. Although pain and palliative services were not explicitly intended for families where older adults age alone, our analysis employing RMIC reflects that their modus operandi, which delivers care at home, has been influential for older adults in migrant households where in‐person care assistance is minimal compared to their counterparts in intergenerational coresidence.

Another emergent systemic barrier is the lack of a grassroots‐level needs assessment, leading to scenarios where the real needs of the beneficiaries are not explored before the commencement of a new programme, resulting in a proliferation of interventions that fail to address unmet needs. Similar findings were observed by Pathare [19], highlighting shortcomings in planning, designing and implementing developmental agendas in aged care. While our analysis unearthed the inadequacies in need assessment, previous evidence on care integration for older adults reported that information blockage and communication gaps co‐exist with perceptual barriers, including mistrust in the system and interprofessional stereotypes in care [49]. In contrast, the absence of trained geriatric professionals, poor integration of social care into primary health, fragmented eldercare policies, and implementation gaps [6, 21, 50, 51] were concluded as the major systemic barriers. Our findings further confirmed the observations of Carbonell et al. [52], who argued that inappropriate fund allocation and utilisation, lack of domain experts in implementation and planning of programmes, and inefficient monitoring systems are the major shortcomings in care planning at a macro level of operation. Thus, despite numerous efforts to care for older adults, substandard care regulations [53] and the hesitation of the target audience to access the services offered continue to reduce the functional efficiency of targeted interventions for older adults in India. This disparity in care coordination calls for urgent sensitisation of both beneficiaries and benefactors to foster quality of alternative care for older people who age alone in India [54].

Besides planning and designing, rigid eligibility criteria to access alternative care are considered a systemic constraint, as encapsulated in RMIC [10]. While previous evidence argues accessibility and affordability as common constraints to care [55], present findings underscore the rigid inclusion criteria where older adults are categorised based on their age and income rather than their functional capabilities, often overlooking the needs that go beyond the instrumental needs and social security in ageing. Furthermore, insights from our analysis further substantiate the demand for a comprehensive framework to integrate long‐term care needs in the nationwide policy responses to ageing, which is a crucial macro‐level care enabler as per RMIC. While the present study highlights the risk of exclusion from alternative care provisions based on age and mobility, Lodha and De Sousa [35] recommended the need to integrate mental health needs and clinical diagnosis into a unified care framework to promote inclusive care for older adults across diverse functional capabilities. Furthermore, our analysis revealed the implementation gap where subject experts with limited field exposure tend to design and implement programmes leading to poor sustainability and contextual feasibility in alternative care delivery. Thus, in line with the existing notion of care barriers, our findings substantiate the absence of contextual frameworks for collaborative care across public and non‐governmental entities in the care economy [3, 19, 39]. In addition to the existing macro‐level barriers, our analysis unearthed political instabilities, dual programmes with overarching objectives and the absence of a comprehensive approach to unify fragmented efforts as macro‐level operational barriers in RMIC. While the insights from this study remain grounded in the socio‐cultural context of Kerala, the findings are often relevant for the LMICs, where similar demographic transitions, caregiving challenges and familial restructuring are observed.

Overall, the findings conclude that a ‘whole‐of‐government’ approach to active ageing and effective age‐friendly governance is often hindered due to the lack of coordination, compartmentalisation and polarisation across disciplines and stakeholders [56], calling for a multidisciplinary effort in strengthening primary healthcare services, geriatric training for healthcare professionals and promoting community‐based care to ensure sustainable care solutions [36]. Thus, our analysis warrants regional, state and central levels of planning, concrete implementation guidelines, participation of older adults in planning and implementation, and efforts to upgrade day care homes into regional resource centres, along with systematic monitoring and evaluation and multi‐stakeholder governance to realise alternative care for older adults.

## 10 Strengths and Limitations of the Study

A key strength of this study lies in its novel approach to exploring the perceived systemic barriers in alternative care from the dyadic perspective across the receiver and provider, using the Rainbow Model of Integrated Approach to capture the care narratives in the South Asian landscape, which have been largely overlooked in the existing research. The present study explored the perceived barriers, challenges and recommendations to strengthen alliterative care from the perspectives of older adults and service providers. Further, data were collected across three geographic regions in Kerala to account for the regional and operational variations in translating alternative care. While the findings offer valuable insights into the systemic barriers, enablers and potential alternatives from older adults and service providers, the scope of the study was limited to older adults who are left behind or stayed behind by choice in the migrant households, and service providers who are primarily involved at an operational level of implementation. Thus, there is ample scope for further investigation into the perception of older adults across other care arrangements and inclusion of service providers at various levels of expertise and experience in care coordination. Furthermore, the present study could not capture the facilitators and barriers at a micro level (familial level), and the perspectives of informal and paid caregivers in adapting the systemic care provisions, warranting further research into the dynamics and realities of intergenerational distance care in migrant households. Further research could explore how diverse care arrangements experience systemic barriers and in what ways public and private integration in care can provide better care avenues for older adults who age alone.

## 11 Conclusion

The study aimed to explore the care realities in migrant households, highlighting the complex interplay between policy regulations, institutional capacities and socio‐cultural determinants that shape care experiences, service providers and receivers. Guided by the Adapted RMIC, our findings reveal enablers in care as well as critical gaps in planning, implementation and service delivery, ranging across micro, meso and macro levels of systemic support, warranting the need for decentralised efforts to bridge the care gap among older adults who age alone in migrant households of Kerala. Care access is often restricted by high costs at the micro level; inadequacy of day care homes at the meso level; and fragile need assessments, poor funding and rigid eligibility norms at the systemic level. Existing barriers in formal care demand a critical shift toward needs‐based and contextually sensitive care models that prioritise inclusivity, sustainability and person‐centred integration across multiple levels of the care continuum. Although there are several care enablers, there is a huge demand for public–private partnerships and community‐based and affordable care choices, which reduce the perceived systemic barriers in social and healthcare services to address the care voids experienced by older adults in migrant households of Kerala. Policies and interventions should transcend beyond clinical practice, building a continuum of care that incorporates the social support that allows older adults to age well at home.

## Author Contributions

**Anu Mohan:** conceptualisation, methodology, software, investigation, data curation, analysis, writing – original draft. **Teddy Andrews Jaihind Jothikaran:** conceptualisation, methodology, writing – review and editing, supervision. **Lena Ashok:** conceptualisation, methodology, writing – review and editing, supervision.

## Ethics Statement

Ethical approval was obtained from the Institutional Ethics Committee (IEC) of Kasturba Medical College (KMC) and Kasturba Hospital (KH), IEC1:248/2022 dated 27 January 2022. All participants were provided with an explanation of the study purpose and procedures, and written informed consent was obtained before participation. The research was conducted in full accordance with the principles of the Declaration of Helsinki [26], ensuring the rights, safety and well‐being of all participants and adhering to all relevant ethical guidelines for research involving human subjects.

## Consent

Written informed consent was obtained from all participants before participation.

## Conflicts of Interest

The authors declare no conflicts of interest.

## Declaration of Generative AI in Scientific Writing

No Generative AI or AI‐assisted technologies were used in the writing process.

## Consent for Publication

No individuals are identifiable in this paper.

## Supporting information


**Supplemental Table 1:** Characteristics table of older adults. **Table 2:** Characteristic Table of service providers.

## Data Availability

Data will be available from the corresponding order upon reasonable request. The datasets generated and analysed during the current study are available from the corresponding author on reasonable request.
