# Supplementary material for: Enablers and Barriers to Alternative Care: Perspectives of Community‐Dwelling Older Adults and Service Providers in Kerala
Source: Health Expect. 2025 Nov 30;28(6):e70507. doi: 10.1111/hex.70507 (PMC12665062; doi:10.1111/hex.70507)
Supplement: Supplementary file 1 — Supplemental Table 1: Characteristics table of older adults. Table 2: Characteristic Table of service providers. [file HEX-28-e70507-s001.docx]

**Supplemental Table 1: Characteristics table of older adults**

| **Participant** | **Age band**  **(in years)** | **Religion** | **Employment status** | **Marital**  **status** | **Medical conditions** | **Health status** | **Living arrangement** | **Number of children** | **Type of migration** | **Availability of a maid or paid caregiver** |
| --- | --- | --- | --- | --- | --- | --- | --- | --- | --- | --- |
| OA-1 | 70-75 | Christian | Returned from Abroad (Private sector) | Widower | Yes | Healthy and mobile | Living alone | 2 | Both internal and international | No |
| OA-2 | 80-85 | Christian | Retired private sector employee | Widower | No | Healthy and partially mobile | Living alone | 2 | International | Yes (full time caretaker) |
| OA-3 | 80-85 | Christian | Retired Public sector employee | Widower | Yes | Partially mobility | Living alone | 4 | Internal | No |
| OA-4 | 75-80 | Christian | Homemaker | Widow | Yes | Healthy and Mobile | Living alone | 1 | International | Yes(full time caretaker) |
| OA-5 | 80-85 | Christian | Retired private sector employee | Widow | No | Healthy and partially mobile | Living alone | 2 | Both | Yes(full time caretaker) |
| OA-6 | 80-85 | Christian | Returned from abroad (Private) | Widow | Yes | Healthy and Mobile | Living alone | 2 | Internal | No |
| OA-7 | 65-70 | Hindu | Retired Private sector employee | Married | No | Healthy and mobile | Living with a spouse | 2 | Both | No |
| OA-8 | 65-70 | Hindu | Homemaker | Married | No | Healthy and mobile | Living with spouse | 2 | Both | No |
| OA-9 | 75-80 | Christian | Retired Public sector employee | Married | Yes | Healthy and mobile | Living with spouse | 2 | both | No |
| OA-10 | 70-75 | Hindu | Retired private sector employee | Married | Yes | Partially mobile | Living with spouse | 2 | Both | No |
| OA-11 | 70-75 | Hindu | Retired Public sector employee | Married | Yes | Healthy and Mobile | Living with spouse | 2 | Both | No |
| OA-12 | 80-85 | Hindu | Retired private sector employee | Widower | Yes | Partially mobile | Living alone | 3 | Internal | Yes (Part-time) |
| OA-13 | 65-70 | Christian | Retired Public sector employee | Married | No | Healthy and mobile | Living with spouse | 2 | Internal | No |
| OA-14 | 65-70 | Hindu | Retired Public sector employee | Married | No | Healthy and Mobile | Living with spouse | 2 | Both | No |
| OA-15 | 65-70 | Hindu | Independent  practitioner | Married | No | Healthy and Mobile | Living with spouse | 2 | Both | No |
| OA-16 | 70-75 | Hindu | Retired public sector employee | Married | No | Healthy and Mobile | Living with spouse | 3 | internal | No |
| OA-17 | 65-70 | Hindu | Home maker | Married | No | Healthy and Mobile | Living with spouse | 2 | Internal | No |
| OA-18 | 65-70 | Christian | Homemaker | Widower | Yes | Healthy and Mobile | Living alone | 2 | International | No |
| OA-19 | 70-75 | Hindu | Retired public sector employee | Married | No | healthy and Mobile | Living alone | 2 | Both | No |
| OA-20 | 70-75 | Christian | Returned from Abroad (Private sector) | Married | No | Healthy and mobile | Living with spouse | 2 | Both | No |

**Table 2: Characteristic Table of service providers**

| **Participant ID** | **Educational Qualification** | **Current role** | **Expert domain/ Summary of major job** | **Key Interventions Undertaken/ Supervised** | **Years of experience** |
| --- | --- | --- | --- | --- | --- |
| SP1 | Post Graduation | State Head -****** | Direct and indirect service designs for older adults across the state. Regulation and supervision of programs run by ***in Kerala | -Mobile health checkups -Model care homes -Help desk for the elderly in hospitals -Livelihood support services -Digital safety campaigns -Disaster management and -Emergency response for older adults | 29 |
| SP2 | SSLC | LSG Representative ******Grama Panchayath, | Direct services with elder care in the ward. | -Identifying and linking beneficiaries with programs run by the panchayath. -Ensuring supply of assistive devices, water bed, medicines, etc, to bedridden beneficiaries | 10 |
| SP3 | Graduation | LSG Representative ******Grama Panchayath | Direct services with elder care in the ward. | Identifying and linking beneficiaries with programs run by the panchayath. | 11 |
| SP4 | SSLC | LSG Representative, ****** Grama Panchayat | Oversee the implementation of development schemes and projects approved by the Panchayat. | -Jagratha Samithi for older adults -Annual and action plan for the panchayath  -Age-friendly local self-governance-related declarations Overview senior citizens' club | 2 |
| SP5 | Post Graduation | Founder -******(Association for Older Adults) and Trainer | Overview of elderly welfare activities in the panchayath In charge of programs under **** | -Monitor and organize ***, a senior citizens club for older adults. -Recreational programs, Regular meetings, Music Club, Mass education campaigns, Support during a pandemic, tele counselling,  -Large-scale survey to create a database for older adults. Pre-palliative care programs. | 8 |
| SP6 | Post Graduation | LSG Representative ******Grama Panchayat | Overview of elderly welfare activities in the panchayath, identification, and linking of older adults with relevant programs by organising them under them | Ward-level support for ***(name of a regional group), identifying beneficiaries who need social support under various schemes | 10 |
| SP7 | Post Graduation | Consultant at****** and Aged care expert | Training of panchayats in various welfare movements | -Training for officials to implement age-friendly LSG initiatives Preparation of handbook and training modules to realise aged care at the grassroots level -Support model initiatives in aged care | 6 |
| SP8 | Graduation | Secretary- ****** and aged care expert | Administrative and coordination role in planning and implementing for older adults | -Empowering older people by addressing their rights and needs. -Consultancy in revising state policies for older people, advocacy, and mass campaigns for increasing pensions, implementation of the Maintenance and Welfare of Parents and Senior Citizens Act, etc. | 14 |

****** name of organization/professional affiliation of the key informant/name of panchayath
